# Gold nanoparticles in pediatric DIPG: promise at the blood-brain barrier’s price

**DOI:** 10.1097/MS9.0000000000005008

**Published:** 2026-04-17

**Authors:** Malaika Zahid, Amina Ahmed, Syed Abdullah Shah, Muhammad Talha, Mahnoor Fatima

**Affiliations:** a Department of Medicine, Sindh Medical College (JSMU), Karachi, Pakistan; b Department of Medicine, King Edward Medical University, Lahore, Pakistan; c Department of Medicine, Shaheed Ziaur Rahman Medical College, Rajshahi Medical University, Bogura, Bangladesh

*Dear Editor,*

Diffuse intrinsic pontine gliomas (DIPG) are childhood malignancies that primarily involve the pons and account for about 300 new cases annually in the US. Its pathogenesis mainly involves H3K27 mutations, resulting from lysine-to-methionine substitution at position 27. They are clinically manifested as the triad of ataxia, pyramidal signs, and cranial nerve palsies with a median survival of 1 year. The conventional therapies remain temozolomide and radiotherapy because surgical resection of the tumor is precluded due to its location, and chemotherapy shows a poor response because of the blood-brain barrier. Hence, targeted agents such as ONC201, CAR T-cell treatments, and nanocarriers are being researched[1].

As only 5% of drugs are capable of crossing the BBB, gold nanoparticles have drawn interest as a targeted delivery technique for neurological diseases (Parkinson’s and Alzheimer’s disease) and brain malignancies. By definition, AuNPs are minute particles (≤100 nm) that exhibit unique physical and chemical properties, making them suitable for biomedical applications. Their carefully controlled size and surface chemistry allow their use as drug carriers across the BBB and promote tumor accumulation through increased permeability and retention effect, enhancing clinical efficacy. Despite great therapeutic promise, their acute toxicity can cause hepatotoxic and cytotoxic consequences, and prolonged use may result in immunoreactivity, inflammation, and accumulations in non-target organs[2].

Experimental trials show that GNPs enhance BBB permeability by interrupting intracellular junctions, disrupting zonula occludens-1, and reducing occludin levels by up to 50–70% in tight junction assays, through mechanisms involving PKCζ phosphorylation^[^3,4^]^, and further promoting siRNA delivery that decreases tumor size in nonclinical models[3], with reported reductions in tumor volume often exceeding 40–60% or achieving multi-fold improvements in delivery efficiency and tumor growth inhibition compared to controls. Recent findings confirm a 26% reduction in target gene expression and a 40% decline in protein abundance, indicating significant penetration of AuNPs across the BBB, highlighting their positive therapeutic effects in glial tumors[5]. Further research reported 54.9% apoptosis in C6 cells and 18.5-fold reduction in IC50 in resistant T98G cells by AuNPs conjugated with temozolomide, as compared to 14.1% with free temozolomide[2].

Studies on nanomedicine show promising short-term efficacy, but they usually ignore long-term safety. By disrupting the integrity of the BBB, AuNPs permit the entry of undesired toxins while causing ultrastructural alterations in the brain. For instance, AuNP exposure in rat models has been shown to increase markers of DNA damage (8-hydroxydeoxyguanosine) by 57%, caspase-3 (apoptosis indicator) by 38%, and heat shock protein 70 (stress response) by 45%. Further evidence shows that AuNPs can cause gold accumulation, which may trigger inflammation, oxidative damage, apoptosis, and sensorimotor dysfunction, ultimately resulting in heavy metal-like neurotoxicity. The developing brain, in particular, is more susceptible to these changes because of non-dividing neurons, high oxygen use, and poor antioxidant response[6]. Besides safety concerns, the lack of established guidelines, batch differences, expensive and complex manufacturing, and ethical concerns may further limit clinical implementations of gold nanoclusters in pediatric DIPG[7].

The treatment complexities for DIPG, despite incremental advances, continue to be a challenge, and this situation accentuates the need for a wise evaluation of the new but underexplored modalities, such as gold nanoparticles, which are very promising. The letter describes existing circumstances that apply to pediatric neuro-oncology nanotechnology and requests dedicated research studies that need to be conducted according to specific safety standards that require testing in relevant preclinical models, which must include models designed for pediatric research. The process requires researchers to establish standardized methods for synthesis and quality control, while creating ethical frameworks that will guide their research activities. Also, they need to create biomarker panels, which will use specific biomarkers to track oxidative stress and apoptosis and stress proteins, including HSP70, for assessing neurotoxicity risks that develop during brain development. This way, the vulnerable young patients could well be protected through the remarkable therapeutic advancements.

## Data Availability

Not applicable to this study.
